# Health-Related Quality of Life in Children with X-Linked Hypophosphatemia Treated with Burosumab: Real-World Data from a German-Swiss Study

**DOI:** 10.1007/s00223-026-01602-x

**Published:** 2026-09-16

**Authors:** Ineke Böckmann, Martin Klein, Helena Mutze, Mirko Rehberg, Giuseppina Spartà, Katrina Evers, Karl Peter Schlingmann, Markus J. Kemper, Annette Richter-Unruh, Dorothee Schmidt, Olaf Hiort, Karina Grohmann-Held, Carmen Schröder, Ute Derichs, Clemens Freiberg, Marcus Weitz, Desiree Dunstheimer, Elmar Schmid, Ulrike John-Kroegel, Oliver Metzing, Sabine Heger, Norbert Jorch, Hagen Staude, Ludwig Patzer, Elke Wühl, Carl-Joachim Partsch, Angela Hübner, Michaela Marx, Christof Land, Norbert Albers, Jens Banzer, Inka Baus, Frauke Wilkening, Kristina Möller, Verena Wagner, Gabriele Krauch, Michaela Gessner, Alexandra Goischke, Maren Leifheit-Nestler, Miroslav Zivicnjak, Dirk Schnabel, Dieter Haffner

**Affiliations:** 1 Department of Pediatric Kidney, Liver, Metabolic and Neurological Diseases, Hannover Medical School, Carl-Neuberg-Str. 1, Hannover, 30625 Germany; 2 Department of Social Sciences, Catholic University of Applied Sciences North Rhine Westphalia, Cologne, Germany; 3 Faculty of Medicine, Department of Pediatrics, University of Cologne, University Hospital Cologne, Cologne, Germany; 4 Pediatric Nephrology, University Children’s Hospital Zurich, Zurich, Switzerland; 5 Department of General Pediatrics, Pediatric Nephrology, University Children’s Hospital, Münster, Germany; 6 Asklepios Children’s Hospital Hamburg-Heidberg, Hamburg, Germany; 7 University Children’s Hospital Bochum, Bochum, Germany; 8 Division of Pediatric Endocrinology and Diabetes, Department of Pediatric and Adolescent Medicine, University of Lübeck, Lübeck, Germany; 9 University Children’s Hospital Greifswald, Greifswald, Germany; 10 University Children’s Hospital, Mainz, Germany; 11 Department of Pediatrics, University Medicine Göttingen, Göttingen, Germany; 12 University Hospital Tübingen, Department of General Pediatrics and Hematology/Oncology, University Children’s Hospital, Tübingen, Germany; 13 Division of Pediatric Endocrinology and Diabetes, Department of Pediatric and Adolescent Medicine, University Hospital of Augsburg, Augsburg, Germany; 14 Pediatric practice Dres. Schmid, Hammon & Zimmermann, Bettendorf, Hirschaid, Germany; 15 Department of Pediatric Nephrology, University Children’s Hospital, Jena, Germany; 16 Department of Pediatric Endocrinology, University Children’s Hospital, Jena, Germany; 17 Kinderkrankenhaus auf der Bult, Pediatric Endocrinology, Hannover, Germany; 18 University Children’s Hospital, Evangelisches Klinikum Bethel, Bielefeld, Germany; 19 Pediatric Nephrology, University Children’s Hospital Rostock, Rostock, Germany; 20 St. Elisabeth and St. Barbara Children’s Hospital, Halle/Saale, Germany; 21 Medical Faculty Heidelberg, Center for Pediatrics and Adolescent Medicine, Division of Pediatric Nephrology, Heidelberg University, Heidelberg, Germany; 22 MVZ Endokrinologikum Hamburg, Hamburg, Germany; 23 Pediatric Endocrinology, University Children’s Hospital, Technical University Dresden, Dresden, Germany; 24 Division of Pediatric Endocrinology, Department Pediatrics, University Hospital of Erlangen, Erlangen, Germany; 25 Center for Pediatric Endocrinology, Gauting, Germany; 26 Department of Pediatric Endocrinology, Christliches Kinderhospital, Osnabrueck, Germany; 27 Division of Pediatric Endocrinology, Department of Paediatrics, Christian-Albrechts- Universität Kiel, Kiel, Germany; 28 Pediatric Nephrology, Department of Pediatric, Helios Clinic Schwerin, Schwerin, Germany; 29 Department of Pediatrics, Klinikum Links der Weser, Bremen, Germany; 30 Pediatric Endocrinology and Diabetology, Pediatric Practice Rostock, Rostock, Germany; 31 Division of Pediatric Endocrinology and Diabetology, Center of Child and Adolescent Medicine, University Medicine, Mannheim, Germany; 32 KfH-Board of Trustees for Dialysis and Kidney Transplantation (KfH-Kuratorium für Dialyse und Nierentransplantation e.V.), Munich, Germany; 33 Nephrology Department, University Children’s Hospital, Basel, Switzerland; 34 Pediatric Endocrinology, ah Center for Chronically Sick Children, University Medicine, Charité Berlin, Germany

**Keywords:** X-linked hypophosphatemia, PHEX, Health-related quality of life, KIDSCREEN-52, Children, Burosumab

## Abstract

Burosumab is effective in improving rickets in children with X-linked hypophosphatemia (XLH). Predictors of health-related quality of life (HRQoL) in pediatric XLH patients treated with burosumab are unknown. In this cross-sectional analysis of a prospective binational observational study, we investigated HRQoL in 64 pediatric XLH patients (36 female) on burosumab treatment, using the KIDSCREEN-52 questionnaire and qualitative interviews. Associations between HRQoL and clinical findings were analysed using multiple regression and receiver operating characteristics (ROC) curve analyses. Median age at investigation and duration of burosumab treatment were 13.1 (IQR 10.4; 14.8) and 2.8 (IQR 1.4; 3.8) years, respectively; 54.7% of patients received secondary burosumab treatment following prior active vitamin D and phosphate. Overall, HRQoL scores were comparable to national reference values. Caregivers rated Physical Well-Being, Moods & Emotions, and Bullying lower than the patients themselves. Burosumab dose was significantly associated with Physical Well-Being, Psychological Well-Being, and Self-Perception in self-report, and with total T-values and Physical Well-Being in proxy questionnaires. The duration of prior treatment with active vitamin D and phosphate was significantly associated with the dimension of Autonomy in self-report and proxy reports. A burosumab dosage above 0.6 mg/kg was associated with a good Physical Well-Being (above the mean of the general population) with a sensitivity and specificity of 75% and 70% in self-report, and 67% and 70% in proxy questionnaires, respectively. In this real-world study, HRQoL in burosumab-treated children with XLH was comparable to that of the general population and associated with burosumab dosage.

**Supplementary Information:**

The online version contains supplementary material available at https://doi.org/10.1007/s00223-026-01602-x.

## Introduction

X-linked hypophosphatemia (XLH) is the most common cause of hereditary hypophosphatemia, with a prevalence ranging from 1.3 to 4.8 cases per 100,000 individuals, and results in a complex metabolic bone disorder [1]. XLH is caused by pathogenic variants in the phosphate-regulating endopeptidase homolog X-linked (*PHEX*) gene, which is predominantly expressed in osteocytes, osteoblasts, and odontoblasts [2, 3]. PHEX malfunction leads to elevated secretion of the phosphaturic hormone fibroblast growth factor 23 (FGF23) from osteocytes and inappropriately high FGF23 levels, resulting in renal phosphate (Pi) wasting via downregulation of the sodium-dependent Pi cotransporters NPT2a and NPT2c, along with reduced synthesis of 1,25-dihydroxy vitamin D (1,25(OH)_2_D) [4]. This ultimately leads to hypophosphatemia and impaired bone mineralization, i.e., rickets (in children) and osteomalacia. XLH usually manifests during the first two years of life, with affected children showing progressive disproportionate short stature, lower limb deformities, and delayed onset of walking [1, 5, 6]. As they age, patients often develop dental abscesses, pseudofractures, hearing loss, enthesopathies, and osteoarthritis, all of which significantly impair quality of life (QoL) and increase adult morbidity [7, 8].

Current guidelines recommend burosumab as the first-line therapy for children with XLH [6, 9]. Burosumab has been shown to have a favourable safety profile and to be superior to the historic treatment with active vitamin D and frequent doses of phosphate salts (active D/Pi) in terms of improving Pi homeostasis and 1,25(OH)_2_D levels and improving rickets [10–12].

As adults with XLH have been shown to experience an impaired health-related QoL (HRQoL) due to pain, functional limitations, anxiety, depression, and social isolation [13, 14], examining HRQoL and identifying potential predictors in children with XLH appears to be of clinical importance. A recent systematic review indicated that burosumab might improve QoL of children with XLH, regarding physical health [9]. An earlier analysis of our binational, prospective, observational study showed that HRQoL in children with XLH was, on average, similar to that of the general population, which may be explained by the fact that the vast majority of patients were switched from active D/Pi to burosumab treatment, as supported by qualitative interviews with the caregivers [15]. Similarly, total HRQoL scores in French children enrolled in the International XLH registry were associated with not taking active D/Pi, and better Physical Health Summary scores with burosumab [16]. However, predictors of HRQoL in children receiving burosumab treatment are largely unknown.

The aim of this study is to identify clinical and biochemical predictors of HRQoL in children with XLH treated with burosumab, in order to integrate these factors into patient management in future. Here we investigated HRQoL, together with key clinical and biochemical parameters in children with XLH treated with burosumab, using the KIDSCREEN-52 questionnaire and qualitative interviews recorded in our binational, prospective, observational study. To account for the age and sex-dependence of parameters of mineral and bone metabolism in children, all biochemical parameters were expressed as age and, where applicable, sex-specific z-scores. The added value of the present study compared with our previous analysis of HRQoL in children with XLH from our cohort [15] lies in its investigation of the associations between the ten distinct HRQoL dimensions and clinical and biochemical factors, aiming to determine whether these findings could have implications for clinical decision-making.

## Materials and methods

### Study design and patients

This study is a cross-sectional analysis of the prospective, binational, multicenter registry and observational study “Growth and comorbidity in children with XLH” by the German Society for Pediatric Nephrology (GPN) and the German Society for Pediatric and Adolescent Endocrinology and Diabetology (DGPAED), which was initiated in 2018 [10]. It was completed according to the declaration of Helsinki and received approval by the Ethics Committee of the Institutional Ethics Review Board at Hannover Medical School (No. 7259), as well as of each participating center. Written informed consent and assent were provided by patients and their parents or guardians. The registry includes German and Swiss patients from birth to 18 years of age who were diagnosed with XLH based on genetic testing and/or typical clinical presentation based on family history, presence of clinical and/or radiological signs of rickets, impaired height velocity and serum Pi levels below the age-related reference range associated with selective renal Pi wasting in the absence of vitamin D or calcium deficiency [6]. From March 2018 until February 2025, 40 participating centers in Germany and Switzerland enrolled 147 XLH patients. In 50 patients (78%), the diagnosis was genetically confirmed. In 4 patients lacking a family history and genetic testing, the diagnosis was made according to characteristic clinical and biochemical features, along with the exclusion of other causes of hypophosphatemic rickets, following the detailed diagnostic approach as recommended [6]. We collected the following clinical data: age, sex, family history, result of *PHEX* gene analysis, age at diagnosis and start of treatment, duration of burosumab and/or active D/Pi treatment, dosage of burosumab treatment, concomitant medication, weight, height, serum Pi, calcium, alkaline phosphatase (ALP), intact parathyroid hormone (iPTH) and 25-hydroxivitamin D (25(OH)D). Biosamples were collected at the respective centers, mostly in the morning, and 7–14 days after the last burosumab injection, as recommended [6]. To ensure temporal consistency, questionnaires and blood samples were collected on the same day during the study visit. Serum values were measured locally. Vitamin D insufficiency and deficiency were defined as a serum 25(OH)D level between 12 and 20 ng/ml and below 12 ng/ml, respectively [17]. Overweight and obesity were defined as BMI > the 90th to ≤ the 97th percentile, and > the 97th percentile, respectively [18]. Physicians followed EMA (European Medicines Agency) recommendations on burosumab treatment in pediatric XLH patients from 2018 [19] and its revised version from 2022 [20]. In the latter, a higher starting dose of burosumab, i.e. 0.8 mg/kg instead of 0.4 mg/kg body weight every two weeks, was recommended. For patients who had initiated burosumab treatment before 2022, the dose was not automatically adjusted to ≥ 0.8 mg/kg but was modified according to clinical requirements like in all other patients, as recommended [6]. Burosumab was administered by trained healthcare professionals.

### KIDSCREEN-52

Quality of life was assessed in the standardized questionnaires KIDSCREEN-52 (child and adolescent version 8 to 18 years for Germany) and the KIDSCREEN-52 (parents version for Germany), consisting of 52 questions across 10 dimensions, i.e. Physical Well-Being (5 items), Psychological Well-Being (6 items), Moods & Emotions (7 items), Self-Perception (5 items), Autonomy (5 items), Parent Relations & Home Life (6 items), Financial Resources (3 items), Peers & Social Support (6 items), School Environment (6 items) and Bullying (3 items). Each dimension has five possible answers, scored from 1 to 5 [21]. In the Financial Resources dimension, children and parents were asked whether the children had enough money in the preceding weeks to buy the things they needed and to participate in the same activities as their friends. All questionnaires were submitted anonymously.

### Qualitative interviews

Based on the dimensions of the KIDSCREEN-52 questionnaire, an open-ended interview guide was developed to capture the multidimensional construct of HRQoL. After obtaining informed consent, telephone interviews of approximately one hour were conducted, recorded, and transcribed. Proxy respondents, all of whom were the patients’ biological parents in this study, were reminded to adopt the perspective of the patient as accurately as possible. Given the limited research on HRQoL in patients with XLH, an open approach was essential to allow the subjective perspectives of patients to emerge. The interviews provided comprehensive insights into life with XLH, enabled clarification of misunderstandings, and facilitated a more precise understanding of complex and individual HRQoL aspects. They also allowed the subjective significance of XLH for quality of life to be assessed and additional influencing factors to be identified. This mixed-methods design enabled a comprehensive, comparable, and individual assessment of HRQoL in this patient group. To preserve participant anonymity, sociodemographic characteristics mentioned in the interview quotations were removed and gender-specific pronouns were supplemented with the corresponding alternative pronoun to avoid disclosure of participants’ gender.

### Statistical analysis

KIDSCREEN-52 questionnaire data were summarized across the ten HRQoL dimensions. T-values were calculated for each dimension and for the total T-value, which summarizes all dimensions, using the KIDSCREEN-52 syntax relying on European reference data, for which the normative mean is 50 and the standard deviation is 10 [21]. T-values are standardized scores with higher scores indicating better HRQoL, except for the Bullying dimension, where higher scored indicate greater problems. For comparison, we used normative data from healthy German children and adolescents aged 8–18 years (*n* = 1,163) and their parents (*n* = 1,658), obtained from the Mental Health Module of the German Health Interview and Examination Survey for Children and Adolescents (KiGGS), conducted by the Robert Koch-Institute [22].

We calculated z-scores using reference values for height and weight [23], BMI [24], serum Pi [25], calcium [26], ALP [27], iPTH [28] and 1,25(OH)_2_D [29], taking age and/or sex into account, where appropriate.

Data are presented as median (interquartile range (IQR)) or n (%), as appropriate. We tested for normal distribution using Shapiro-Wilk tests. We used the Mann-Whitney-U test or the Chi-squared test to verify differences between the groups, as appropriate. We used two-sided one-sample Wilcoxon signed-rank tests to assess whether the median T-values of each HRQoL dimension and z-scores of clinical and biochemical parameters of our cohort differed significantly from the respective normative values. We conducted Spearman’s Rho correlation analyses with a threshold of *p* < 0.10 and subsequent multiple regression analyses, to identify associations between T-values of the HRQoL dimensions and potential predictors, including age, age at diagnosis and start of burosumab, sex, duration of burosumab and prior treatment with active D/Pi, dosage of burosumab, height, weight and BMI z scores, z-scores of serum Pi, Ca, iPTH, 1,25(OH)_2_D and ALP, vitamin D status (vitamin D sufficiency versus insufficiency or deficiency), hypophosphatemia (serum Pi < -2.0 z-score), elevated ALP (ALP z-score > 2.0) and the T-values for the Financial Resources dimension of the KIDSCREEN-52. To investigate whether clinical aspects could discriminate between patients with good and poor quality of life, receiver operating characteristics (ROC) curves were generated. Quality of life was classified as “good” or “poor” according to the median German pediatric reference values for each of the ten HRQoL dimensions (good = T-value equal to or above the mean of the reference population; poor = T-values below the mean of the reference population). The area under the curve (AUC) was calculated to assess the discriminatory power of medication dose as a predictive parameter, with values of 0.5 and 1.0 indicating none and perfect discrimination, respectively. The optimal cut-off value for the burosumab dose for differentiating between the two groups of “good” and “poor” HRQoL was determined using the Youden index, which identifies the cut-off point with the highest sum of sensitivity and specificity. *p* values < 0.05 were considered statistically significant.

SPSS Statistics for Mac, version 29.0.2.0 (IBM Corporation, Armonk, NY, USA) and GraphPad Prism for Mac, version 10.4.1 (GraphPad Software, Boston, MA, USA) were used.

## Results

### Patient characteristics

From the 147 patients enrolled in our binational registry, 80 patients met the inclusion criteria for this cross-sectional analysis of being between 8 and 18 years of age, allowing use of the KIDSCREEN-52 questionnaires, and receiving treatment with burosumab. Of these, 64 patients (80%), all from Germany, had available KIDSCREEN-52 questionnaires. KIDSCREEEN-52 self-report questionnaires were obtained from 49 patients (76.6%), proxy questionnaires from 59 patients’ parents (92.2%), and both self-report and proxy questionnaires from 43 patients (67.2%). At least one of the two questionnaires was obtained from each patient included. Telephone interviews were conducted with 21 patients. 36 of the 64 patients included were female and median age was 13.1 years (IQR 10.4; 14.8) (Table 1). Age at diagnosis was 1.9 years (IQR 0.2; 0.3) and 0.2 years (IQR 0.0; 2.0) in girls and boys, respectively (*p* < 0.05). In 5.6% of female and 28.6% of male patients, a positive family history was known (*p* < 0.05). At the time of investigation, patients were treated with burosumab at a median dose of 0.63 mg/kg per two weeks (IQR 0.36;1.12) for a median duration of 2.8 years (IQR 1.4; 3.8). 54.7% of patients had received prior treatment with active D/Pi for a median period of 7.2 years (IQR 4.6; 9.9). Patients’ median height and weight were below the German reference range (-1.92 and − 0.64 z-score, respectively), while BMI was elevated (0.64 z-score; each *p* < 0.05), with 13% and 17% of patients classified as overweight or obese, respectively.

**Table 1:** Clinical characteristics of 64 pediatric XLH patients on burosumab treatment

Characteristics	All	Female	Male	*p*
n (%)	64 (100)	36 (56.3)	28 (43.7)	n.a.
Age at diagnosis, years	1.0 (0.06; 3.0)	1.9 (0.2; 3.0)	0.2 (0.0; 2.0)	**0.016**
Positive family history, n (%)	10 (15.6)	2 (5.6)	8 (28.6)	**0.012**
Age at observation, years	13.05 (10.39; 14.79)	12.0 (10.5; 14.6)	13.6 (10.3; 15.1)	0.598
Age at start of burosumab, years	9.8 (7.6; 12.2)	9.8 (8.0; 11.9)	10.2 (8.4; 12.2)	0.675
Duration burosumab, years	2.78 (1.36; 3.79)	2.43 (1.29; 3.47)	2.27 (1.21; 3.87)	0.749
Burosumab dosage, mg/kg^d^	0.63 (0.36; 1.12)	0.73 (0.48; 1.13)	0.58 (0.31; 1.07)	0.166
Prior treatment active D/Pi, n (%)	35 (54.7)	22 (61.1)	13 (46.6)	0.460
Prior treatment active D/Pi, years	7.2 (4.6; 9.9)	7.2 (4.2; 9.6)	9.1 (3.3; 12.5)	0.415
Height, cm	146.1 (131.2; 153.6)	141.6 (130.2; 152.5)	147.4 (130.3; 154.3)	0.548
Height, z-score	− 1.92 (− 2.71; − 1.17) ^c^	− 1.95 (− 2.73; − 0.61) ^c^	− 1.98 (− 3.04; − 1.46) ^c^	0.325
Weight, kg	47.9 (32.2; 55.5)	43.6 (32.5; 54.1)	47.2 (29.0; 52.9)	0.543
Weight, z-score	− 0.64 (− 1.50; 0.49) ^b^	− 0.78 (− 2.07; 0.35)	− 0.91 (− 1.49; − 0.46) ^a^	0.855
BMI, kg/m	20.69 (18.06; 24.46)	20.6 (17.6; 24.0)	20.2 (18.1; 24.2)	0.598
BMI, z-score	0.64 (0.005; 1.49) ^c^	0.53 (− 0.29; 1.48) ^a^	0.61 (0.06; 1.27) ^c^	0.481
Overweight, n (%)	8 (12.5)	5 (13.9)	3 (10.7)	0.703
Obesity, n (%)	11 (17.2)	6 (16.7)	5 (17.9)	0.900
Self-report questionnaires, n (%)	49 (76.6)	29 (80.6)	20 (71.4)	0.393
Proxy questionnaires^e^, n (%)	59 (92.2)	34 (94.4)	25 (85.7)	0.446
Both Proxy & Self-report, n (%)	43 (67.2)	27 (75.0)	16 (57.1)	0.131

Data is presented as median (interquartile range) or n (%), *p* values were calculated using one sample Wilcoxon test, unpaired Mann-Whitney test and Chi-Pearson-Square test, as appropriate. ^a^ *p* < 0.05; ^b^ *p* < 0.01; ^c^ *p* < 0.001 versus healthy controls; ^d^ given every two weeks; Active D/Pi, active vitamin D and phosphate salts;

^e^ information provided by biological parents; BMI, body mass index

Bold significance levels indicate a p value < 0.05

### Parameters of mineral and bone metabolism

Patients had a reduced median serum Pi (− 2.44 z-score, *p* < 0.01), increased ALP (1.46 z-score, *p* > 0.001), and increased iPTH (1.11 z-score, *p* < 0.001) compared to healthy children, while median serum Ca and 1,25(OH)D z-scores were within the normal range (Table 2). Vitamin D deficiency or insufficiency was noted in 9/64 (14%) of patients.

**Table 2:** Parameters of mineral and bone metabolism in 64 pediatric XLH patients on burosumab treatment

Characteristics	All
Serum phosphate, mmol/l	0.97 (0.87; 1.10)
Serum phosphate, z-score	− 2.44 (− 3.05; − 1.53) ^a^
Serum ALP, U/l	318 (250; 417)
Serum ALP, z-score	1.46 (0.33; 2.95) ^a^
Serum calcium, mmol/l	2.40 (2.35; 2.47)
Serum calcium, z-score	− 0.45 (− 1.81; 0.59)
Serum iPTH, ng/l	44.3 (34.0; 62.6)
Serum iPTH, z-score	1.11 (0.39; 2.05) ^a^
Serum 1,25(OH)_2_D, pg/ml	73.8 (51.6; 84.1)
Serum 1,25(OH)_2_D, z-score	0.62 (− 1.11; 1.26)
Serum 25(OH)D, ng/ml	22.6 (19.4; 26.7)
Vitamin D sufficiency, n (%)	55 (86)

Data is presented as median (interquartile range) or n (%), *p* values were calculated using one sample Wilcoxon test and unpaired Mann-Whitney test, as appropriate. ^a^ *p* < 0.05 versus healthy children. AP, alkaline phosphatase; iPTH, intact parathyroid hormone; 25(OH)D, 25-hydroxyvitamin D; 1,25(OH)2D, 1,25-dihydroxyvitamin D

### HRQoL rated by patients and caregivers

**Fig. 1:**
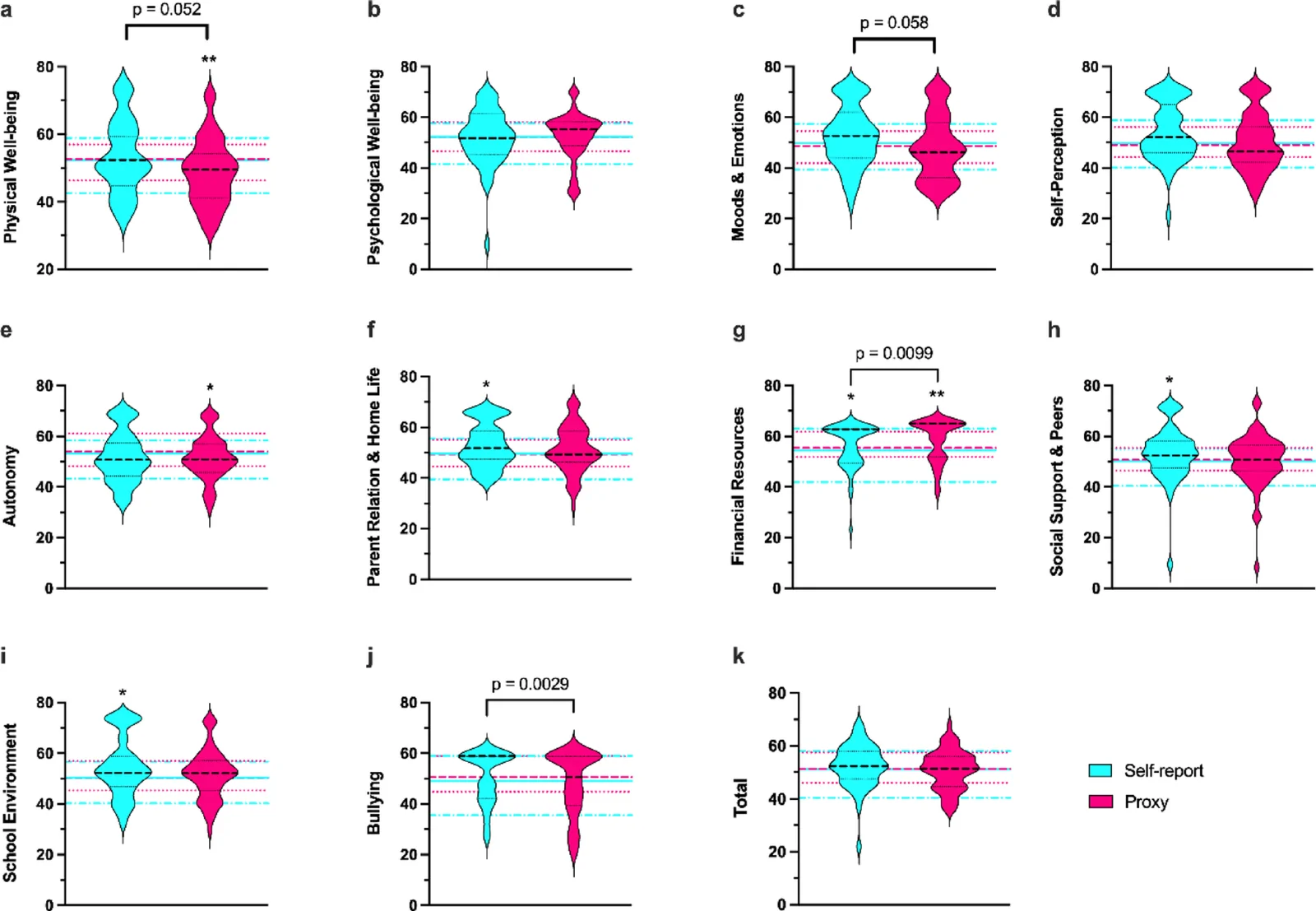
T-values of 10 dimensions of HRQoL in children with XLH treated with burosumab from self-report (*n* = 49) and proxy (*n* = 59) questionnaires. Data are given as T-values in the ten dimensions of HRQoL according to KIDSCREEN-52. Colored lines indicate the median, the 25th and 75th percentiles of the respective German reference values for self-report and proxy questionnaires. *p < 0.05; **p < 0.001

In self-report questionnaires, HRQoL scores were comparable to or even higher than German reference values (Fig. 1, Supplementary Table 1). Children with XLH in our cohort rated their Parent Relation & Home Life, Financial Resources, Peers & Social Support, and School Environment higher than the reference values (each *p* < 0.05). In proxy questionnaires, most of the dimensions were also comparable to the reference values apart from lower scores regarding Physical Well-Being (*p* < 0.01) and Autonomy (*p* < 0.05), and higher scores regarding Financial Resources (*p* < 0.001).

Some of the HRQoL dimensions were rated differently by patients and their caregivers (Fig. 1). While Financial Resources were rated higher (*p* < 0.01), Bullying was rated lower (*p* < 0.01) by the caregivers. Physical Well-Being and Moods & Emotions also tended to achieve a lower score in proxy than in self-report questionnaires, although this did not reach a level of statistical significance (*p* = 0.052 and *p* = 0.058). There were no significant differences between female and male patients regarding the scores of self-report and proxy questionnaires on HRQoL (Supplementary Tables 2 and 3).

### Bivariate correlations of HRQoL scores and clinical findings

**Fig. 2:**
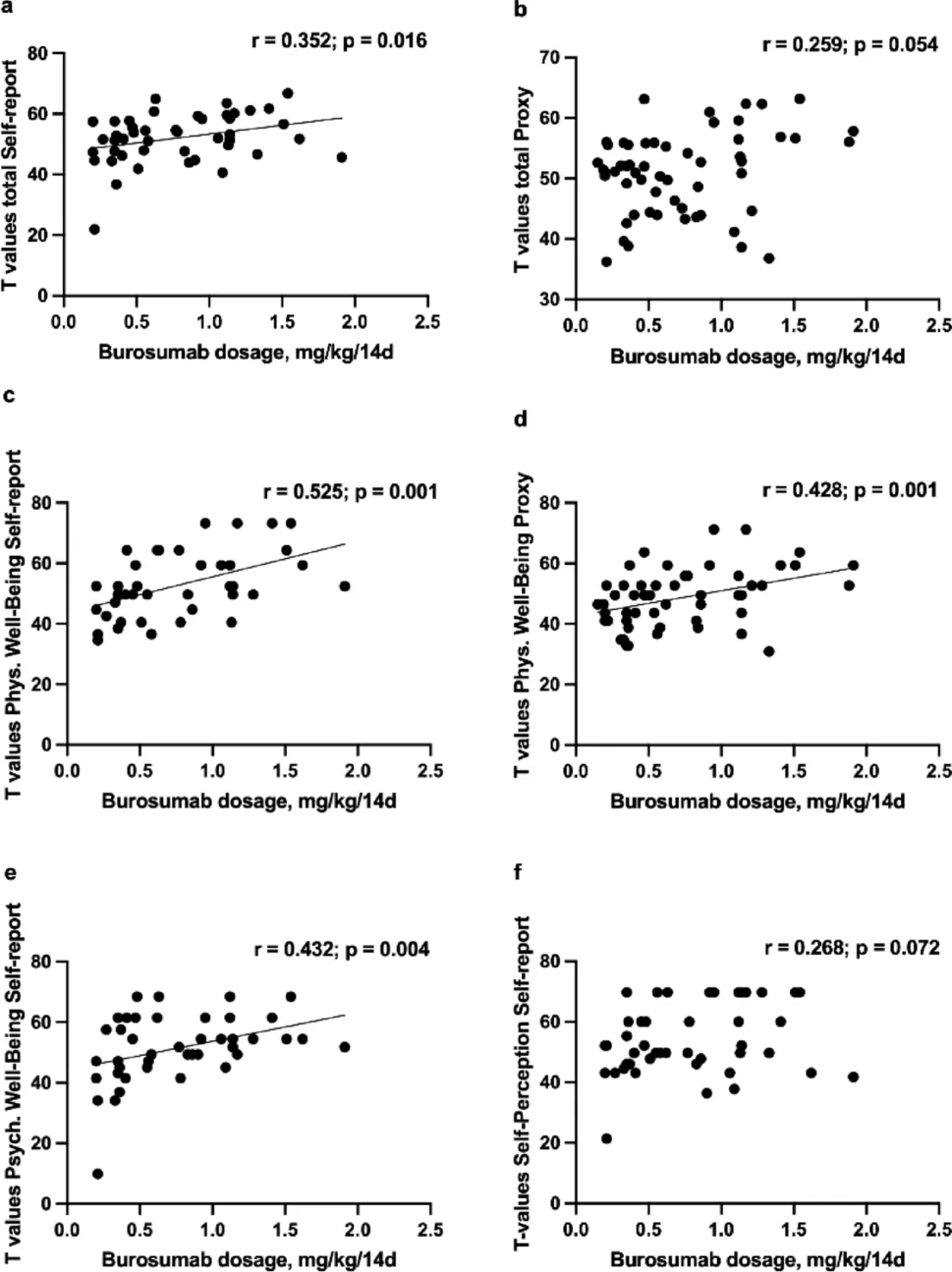
Associations between HRQoL scores and burosumab dosage from self-report and proxy questionnaires. r, Spearman’s correlation coefficient; mg/kg/14d, mg per kg body weight administered every 14 days; Phys, physical; psych, psychological

**Fig. 3:**
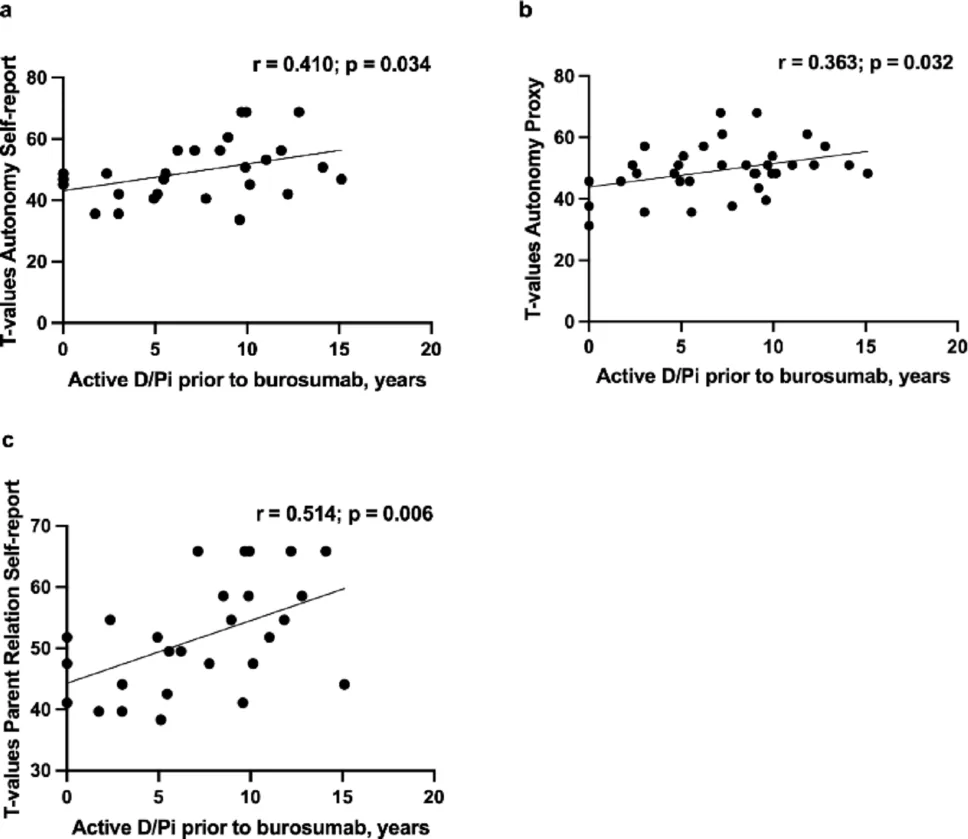
Associations between HRQoL scores and duration of prior treatment with active vitamin D and phosphate from self-report and proxy questionnaires. r, Spearman’s correlation coefficient; active D/Pi, active vitamin D and phosphate salts

In Spearman’s correlation analysis, serum ALP z-score was negatively associated with Psychological Well-Being (*r* = − 0.342; *p* < 0.05) in self-report questionnaires. Intact PTH z-score was associated with Self-Perception (*r* = 0.381; *p* < 0.05) and Financial Resources (*r* = 0.412; *p* < 0.05) in self-report questionnaires. In proxy questionnaires, calcium z-score was negatively associated with Self-Perception (*r* = − 0.361; *p* < 0.05). Regarding clinical aspects, age was negatively associated with Physical Well-Being (*r* = − 0.323; *p* < 0.05) and positively with poor Social Acceptance (Bullying) (*r* = 0.370; *p* < 0.05), BMI z-score was negatively associated with poor Social Acceptance (*r* = − 0.365; *p* < 0.05) and total T-values (*r* = − 0.303; *p* < 0.05) and overweight was associated with School Environment (*r* = 0.356; *p* < 0.05) in self-report questionnaires. Burosumab dosage was significantly associated with total T-values (*r* = 0.352, *p* < 0.05), Physical Well-Being (*r* = 0.525, *p* < 0.01), and Psychological Well-Being (*r* = 0.432, *p* < 0.001) in self-report, and Physical Well-Being in proxy (*r* = 0.428, *p* < 0.01) questionnaires (Fig. 2), while associations of burosumab dosage with total T-values in proxy (*r* = 0.259, *p* = 0.054) and Self-Perception in self-report (*r* = 0.268, *p* = 0.072) questionnaires did not reach a level of statistical significance. The duration of active D/Pi treatment prior to burosumab was associated with Autonomy in both, self-report (*r* = 0.410, *p* < 0.05) and proxy (*r* = 0.363, *p* < 0.05), and with the dimension of Parent Relation (*r* = 0.514, *p* < 0.01) in self-report questionnaires (Fig. 3).

### Predictors of HRQoL scores in the multivariable analysis

The dimensions of HRQoL recorded in the self-report questionnaires were associated with clinical findings and T-values of Financial Resources in the multivariable analysis (Table 3). Physical Well-Being was associated with burosumab dosage, and T-values of Financial Resources (cumulative r^2^ = 0.387). Psychological Well-Being was associated with burosumab dosage and T-values of Financial Resources (cumulative r^2^ = 0.281). Self-Perception was associated with burosumab dosage and T-values of Financial Resources (cumulative r^2^ = 0.434). In the multivariable regression analysis the association between Psychological Well-Being and the ALP z-score was no longer statistically significant. Autonomy and Parent Relation & Home Life were associated with the duration of treatment with active D/Pi prior to burosumab (r^2^ = 0.158 and 0.249; respectively). School Environment was associated with overweight (r^2^ = 0.093). Bullying was positively associated with age and negatively with BMI z-score (cumulative r^2^ = 0.510).

**Table 3:** Multiple linear regression models of variables associated with HRQoL scores from self-report and proxy questionnaires in pediatric XLH patients treated with burosumab

Self-report questionnaires	Physical well-being		Psychological well-being		Self-perception		Autonomy		Parent relation & home life		School environment		Bullying	
	β	*p*	β	*p*	β	*p*	β	*p*	β	*p*	β	*p*	β	*p*
Age, years													0.557	0.001
BMI, z-score													− 0.493	0.003
Overweight											0.305	0.047		
Burosumab dosage, mg/kg^a^	0.493	0.001	0.415	0.011	0.420	0.010								
Prior active D/Pi, years							0.397	0.040	0.499	0.008				
T-value Financial Resources proxy	0.344	0.017	0.316	0.047	0.510	0.002								
R² of the regression model	0.387		0.281		0.434		0.158		0.249		0.093		0.510	

Proxy questionnaires^b^	Total		Physical well-being		Autonomy		Peers & social support	
	β	*p*	β	*p*	β	*p*	β	*p*
Burosumab dosage, mg/kg^a^	0.211	0.049	0.367	0.004				
Duration burosumab, years							0.311	0.005
Prior active D/Pi, years					0.381	0.029		
T-value Financial Resources proxy	0.619	< 0.001	0.310	0.015			0.573	< 0.001
R² of the regression model	0.447		0.254		0.145		0.434	

The analysis of the proxy questionnaires also revealed several associations with clinical factors in the multivariable analysis (Table 3). Total T-values were associated with burosumab dosage and T-values of Financial Resources (cumulative r^2^ = 0.447). Physical Well-Being was associated with burosumab dosage and T-values of Financial Resources (cumulative r^2^ = 0.254). Autonomy was associated with prior active D/Pi treatment (r^2^ = 0.145). Peers & Social Support was associated with the duration of burosumab treatment and T-values of Financial Resources (cumulative r^2^ = 0.434). In contrast, all other potential predictors, including age at diagnosis and start of burosumab, sex, and z-scores for height, weight, serum Pi, Ca, ALP, iPTH, and 1,25(OH)_2_D, and vitamin D sufficiency, as well as hypophosphatemia or elevated ALP were not significant correlates of HRQoL scores in each of the 10 dimensions of HRQoL from self-report or proxy questionnaires in the multivariable analysis.

### Association of burosumab dosage with HRQoL scores in ROC curve analysis

**Fig. 4:**
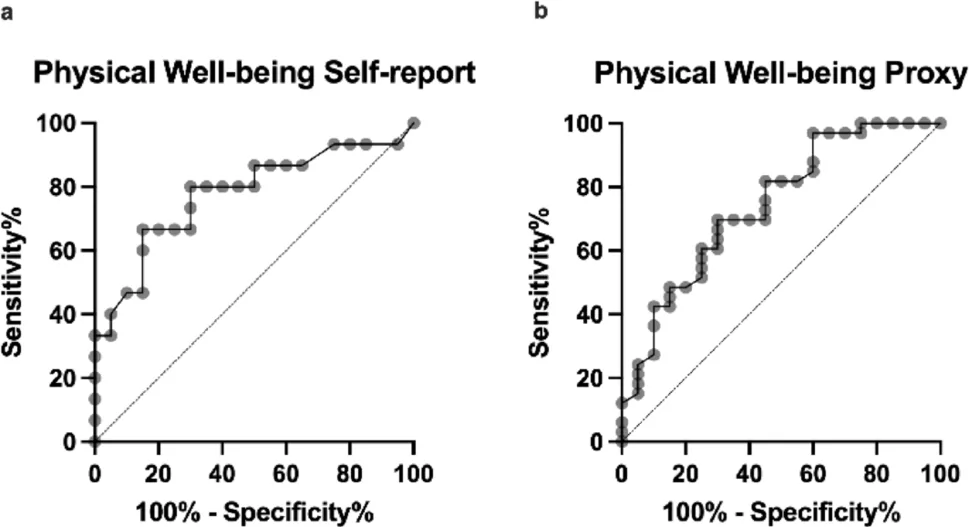
Receiver operating characteristic (ROC) curves examining burosumab dosage as a predictor of HRQoL. The cut-off between good and poor HRQoL was defined as T-values above or below the German reference values in each dimension displayed. **a**: AUC = 0.777 (95% CI 0.611–0.942; *p* = 0.006), optimal cut-off value 0.5993 mg/kg/14d (sensitivity 75.0%; specificity 70.0%) **b**: AUC = 0.744 (95% CI 0.606–0.882; *p* = 0.003), optimal cut-off value 0.6253 mg/kg/14d (sensitivity 66.8%; specificity 69.7%)

To further evaluate the predictive value of burosumab dosage for a good HRQoL in the dimension of Physical Well-Being in the self-report questionnaires, ROC analysis was performed demonstrating an area under the ROC curve (AUC) of 0.777 (95% CI 0.611–0.942, *p* = 0.006), indicating a good discrimination of T-values above or below the mean of German healthy children (Fig. 4). The optimal cut-off value, determined using the Youden index, was 0.5993 mg/kg every 2 weeks, corresponding to a sensitivity of 75.0% and a specificity of 70.0%. Burosumab dosage also showed good discriminatory ability regarding Physical Well-Being scores in proxy questionnaires (AUC = 0.744, 95% CI 0.606–0.882, *p* = 0.003, optimal cut-off 0.6253 mg/kg every 2 weeks with 66.8% sensitivity and 69.7% specificity).

### Comparison of biochemical parameters of mineral and vitamin D metabolism between patients treated with a burosumab dosage below and at least 0.6 mg/kg given every 14 days

Patients receiving a burosumab dose of less than 0.6 mg/kg every 14 days showed slightly lower median Pi and higher ALP and iPTH z-scores compared to those receiving higher burosumab doses. However, these differences did not reach the level of statistical significance (Supplementary Table 4, Supplementary Fig. 1).

### Qualitative interviews

Qualitative telephone interviews were conducted with 21 patients or caregivers. The HRQoL dimensions mentioned above were assessed. Key quotes from the interviews are listed in Table 4. The content of the interviews is outlined in detail in conjunction with the quantitative analysis of HRQoL in the discussion section.

**Table 4:** Results of health-related quality of life assessed by qualitative interviews

Dimension	Qualitative interviews - quotation
Physical well-being	‘Mainly pain in the legs or hips.’ (004_002 (2) 25:04) ‘Yes, she/he often has a little pain. Especially in the lower extremities. So feet, ankles. Yes, that’s basically it. So ankles and feet.’ (002_005 (1) 38:41) ‘Okay, she/he can’t walk 30 kilometers now, but if she/he has walked five or six kilometers, then she/he can cope with it well in the evening. Then later she/he gets what I always call bone pain. It’s always difficult to interpret because you can’t put yourself in her/his shoes. But she/he doesn’t have any muscular problems, it’s her/his bones that cause her/him problems.’ (001_001 (1) 12:14)
Psychological well-being	‘To be happy and not have so many health problems. That worries her/him from time to time.’ (001_001 (1) 02:16) ‘It’s sometimes hard to describe. I can’t say exactly, so yes and no at the same time. Sometimes I can’t say exactly what it is, but sometimes I just feel sad because I feel sad. As stupid as that sounds.‘ (004_002 (1) 32:08)
Moods and emotions / self-perception	‘I don’t think she/he sees it as such a big deal her-/himself. Sure, she/he can’t reach everything now, but that’s not a limitation for her/him. It’s just that everyone else seems to have a problem with it.’ (002_003(1), 01:27:21). ‘She/he doesn’t feel well. Not at all. No. She/he has… no… she/he feels very uncomfortable. The thing is, she/he has very severe bow legs due to her/his illness, and that’s an absolute nightmare for her/him. It’s the looks she/he gets from people on the street and, the comments they make. That’s really awful. (…) She/he actually finds herself disgusting and stuff. So it’s really bad. Exactly. But, it’s, I don’t know if that, well, I’ll just say it’ (010_001 (1), 01:37) ’How is her/his [quality of life] right now?! Well, not very high. She/he doesn’t have much quality of life, I think, I feel.’ (010_001 (1), 05:32)
Autonomy	,So it’s definitely much more relaxed to get an injection every two weeks and just have to be at home than to have to carry a whole freezer-thing around with you every time. Or a refrigerator or something, so that you always have the medication with you. So I have a lot more time for friends and family.’ (031_001, 05:40). ,Before, we always had to make sure that we were there at the right times and had our medication with us. At school, we sometimes had to be taken out of class to go to the staff room and take our medication there, because that was the only place with a refrigerator. So now things are much more relaxed.’ (031_002, 10:59). ,But she/he also says that her/his quality of life has improved significantly thanks to burosumab in relation to phosphate diabetes. […] She/he also used to take monosodium phosphate, which was very stressful. So if we had to talk about that, her/his quality of life would be significantly lower. […] It was really stressful to have to take monosodium phosphate every two hours, no matter where you were. And always being asked, ,Have you taken it?’’ (003_002 (1), 05:52)
Peers & social support	‘So friends, hobbies, and family. She/he is well taken care of there. […] Her/his best friend [very important]. […] Well, I think so, we are [a great support], and the dog is also an issue at the moment. She/he also says that he is incredibly important to her/him. Yes, it’s such an important time. That someone else is there and doesn’t say anything.’ 002_002 (1) 36:46 ‘That’s absolutely unchanged, because she/he is a people person. And for her/him, it’s really important to maintain relationships. And because of that, her/his social circle is still the same. And also the intensity. So that’s unchanged. At some point, girls/boys will join the picture. Not yet.’ 003_002 (3) 30:51
School Environment	‘Because she/he is such a sensitive child, she/he has his close friends, some of whom she/he has known since she/he was a baby through friends of ours. She/he already has a few school friends, but they’re all kindergarten friends who have stayed in touch and are the most important to her/him. Kindergarten friends and friends from the neighborhood. And then she/he has his 1, 2, 3 friends at school, but they’re not that relevant. What’s really relevant are her/his old friends, who have all grown up with her/him and who naturally give her/him a tremendous amount of security. And even if there’s something she/he can’t do, it’s not an issue for anyone.’ 003_002 (1) 30:55 ‘Yes, she/he does her/his sports club at school once a week. And then she/he rides her bike and scooter around the neighborhood with her/his friends from time to time. And we went to the swimming pool, where she/he was active the whole time.’ 032_003 (2) 19:28
Social acceptance (bullying)	‘Well, exactly, and the concern, my husband just said, we do have a bit of bullying, of course. There have also been a few incidents, which she/he doesn’t tell us about, but we’ve heard from others, where children have teased her/him a little. That’s what worries us, of course.’ (032_003(1), 09:13)

## Discussion

This is one of the first studies to systematically and quantitatively investigate associations of HRQoL from self-report and proxy questionnaires with clinical parameters in children with XLH treated with burosumab. It is worth noting that in Germany and Switzerland, burosumab is covered by both public and private health insurance, and the prescribed dose of burosumab is determined solely by the treating physician. Overall, the HRQoL in our pediatric XLH cohort treated with burosumab for a median period of 2.8 years was comparable to or slightly higher than the German pediatric reference population [22]. In self-report questionnaires, children with XLH rated the dimensions of Parent Relation & Home Life, Financial Resources, Peers & Social Support, and School Environment higher than the German reference values. In proxy questionnaires, caregivers rated Financial Resources above the German national average.

The dimension of Autonomy achieved lower scores in proxy questionnaires than in the German reference values. This is in line with the well-known tendency of parents of children with chronic illnesses to perceive their child as more vulnerable and less autonomous than parents of healthy peers [30, 31]. As it is known that higher perceived child vulnerability in chronically sick children might lead to overprotective parenting and psychosocial problems [32], parents should be encouraged to foster their children’s autonomy and avoid overcontrol.

This is the first study to compare self-reported and proxy-reported HRQoL in children with XLH. Our findings indicate differences between children’s self-perceptions and their parents’ assessments. The dimension of Physical Well-Being achieved lower scores in proxy than in self-report questionnaires but just missed statistical significance (*p* = 0.052). One possible explanation could be that parents perceive their children’s limitations in keeping up with their peers in everyday physical activities more realistically, i.e. as more severe or burdensome than the children themselves [33, 34]. A corresponding quotation from our telephone interviews illustrates this point: ‘I don’t think she/he sees it as such a big deal her-/himself. Sure, she/he can’t reach everything now, but that’s not a limitation for her/him. It’s just that everyone else seems to have a problem with it.’ (Table 4). In proxy questionnaires, the dimension of Moods & Emotions tended to achieve lower T-values than in self-report questionnaires (*p* = 0.058). This may reflect that most children in our cohort were experiencing puberty at the time of assessment, and parents tend to underestimate the emotional well-being of their pubertal children [35, 36]. This finding is supported by quotations from a telephone interview in the dimension Moods & Emotions in Table 4. Bullying was also rated lower in proxy than in self-report questionnaires (*p* = 0.003). Evidence from the literature suggests that parents may be more sensitive than their children to teasing or negative social feedback, both neurobiologically and emotionally [37]. A statement reflecting this perspective is given in the dimension Social Acceptance (Bullying) in Table 4. Financial Resources were rated higher by proxy than by self-report questionnaires. It is reasonable to assume that parents have a more accurate understanding of their household income than their children.

In the multivariable analysis, the duration of prior active D/Pi treatment was associated with HRQoL in the dimension of Autonomy in both self-report and proxy, as well as with Parent Relation & Home Life in self-report questionnaires. This may be explained by the reduced treatment burden under burosumab treatment, requiring only one subcutaneous injection every 14 days replacing multiple daily phosphate doses, combined with children’s reluctance to take oral medication especially during adolescence. This may also present a “honeymoon effect”, which may diminish in the following years. This could be supported by the fact that the dimension of Peers & Social Support in proxy reports is associated with the duration of burosumab treatment. This finding is exemplified by the quotations from telephone interviews in the HRQoL dimension Autonomy in Table 4.

Multivariable analysis revealed associations of burosumab dosage with HRQoL scores of Physical Well-Being, Psychological Well-Being and Self-Perception in self-reports, as well as with total T-values and Physical Well-Being in proxy questionnaires. The observation that there is an association of burosumab dosage with Physical Well-Being in both self-report and proxy questionnaires may support the credibility of this link. There is supporting evidence in the literature reporting better physical functioning and reduction of pain in pediatric and adult XLH patients treated with burosumab compared to active D/Pi [11, 38, 39] and stating the correction of lower limb malalignment when switching treatment from active D/Pi to burosumab [40]. It could be assumed that the association between burosumab dosage and Psychological Well-Being and Self-Perception may, at least partly, stem from an improvement in Physical Well-Being achieved with adequate burosumab doses as physical health and activity are known to enhance overall quality of life [41, 42].

Burosumab dosage showed good discriminatory ability for Physical Well-Being in both self-report (AUC = 0.777), and proxy questionnaires (AUC = 0.744) and optimal cut-off values for a good HRQoL of 0.60 mg/kg and 0.63 mg/kg, respectively. This is slightly below the current EMA recommended starting dose (0.8 mg/kg), but above the recommended starting dose up to 2022 (0.4 mg/kg). The sensitivity of the identified cut-off suggests patients receiving burosumab above this dose to have Physical Well-Being HRQoL scores above the German average. Conversely, the specificity suggests that patients receiving burosumab below this cut-off are more likely to have scores below the German average for Physical Well-Being. However, the burosumab dose required for symptom control appears to vary individually, which is also evident from the wide range of dosages observed among our patients (0.2–1.9 mg/kg/14d) and in previous real-world studies in children with XLH [10, 43–45]. Of note, the majority of patients (66%) showed ALP z-scores below the upper limit of normal, suggesting good control of rickets activity. The high variability in burosumab dosage may therefore reflect the high phenotypic variability of the disease in patients treated outside clinical trials [10, 43]. Indeed, in the phase 3 registration trial for burosumab, only children with XLH showing a high radiological rickets severity score of 2.0 or higher were enrolled [11], despite prior treatment with adequate doses of active D/Pi.

Surprisingly, our multivariate analysis of clinical predictors of HRQoL in children with XLH receiving burosumab treatment revealed that none of the mineral and bone metabolism parameters remained significant in the final regression model. Only burosumab dosage emerged as an independent predictor of HRQoL. Additionally, there were no significant differences in parameters of mineral and vitamin D metabolism when comparing patients receiving a burosumab dose below 0.6 mg/kg every 14 days with patients receiving burosumab equal or above this threshold. This contrasts with the recent findings of Linglart et al., who reported an association between quality of life and absolute serum Pi levels among children treated with either burosumab or active D/Pi enrolled in the International XLH Registry [16]. This discrepancy may, at least in part, be attributable to methodological differences: the previous study relied on bivariate analysis and absolute serum Pi levels, whereas the present study employed multivariable analysis and age- and sex-adjusted z-scores for serum Pi, which account for physiological variations during growth and development. Since the ALP levels of the vast majority of patients were already below the upper limit of normal, our results can also be interpreted to suggest that the complex effects of burosumab on HRQoL cannot be explained by individual laboratory parameters alone and that a minimum effective dose of burosumab is likely required to achieve good HRQoL, beyond biochemical normalization.

Interestingly, female patients were significantly older at the time of diagnosis than male patients (1.9 vs. 0.2 years, *p* < 0.05). This discrepancy could at least partly be explained by the higher proportion of a known positive family history in boys (28.6% vs. 5.6% in girls) which likely increased awareness of the disease among both families and clinicians.

The present study has several limitations. First, the patient cohort is relatively small, and not for all participants both self-report and proxy questionnaires were available. However, every patient provided at least one version. Second, our cohort is relatively homogeneous with respect to treatment initiation. Since early initiation of XLH therapy has been reported to be associated with a better clinical course and quality of life [46, 47], this may have limited the ability to distinguish the effects of individual clinical factors on HRQoL. Third, we cannot determine the extent to which HRQoL improves when patients switch from active D/Pi to burosumab, as the German-Swiss XLH Registry is an observational study, and the vast majority of pediatric XLH patients were already switched to burosumab treatment before enrollment. Therefore, we do not have sufficient data on HRQoL from the period prior to the start of treatment with burosumab (*n* = 8) to perform a meaningful statistical analysis. Fourth, we cannot exclude the possibility that expectation-related or placebo-like effects contributed to the association between higher burosumab doses and better self- and proxy-reported Physical Well-Being. Finally, families in our cohort appear to have above-average Financial Resources. This could be a confounder regarding all other dimensions of HRQoL, as an above-average socio-economic status is known to be associated with a better quality of life of children with chronic disease [48]. In the multivariable analysis of this study, the T-values in the dimension of Financial Resources were shown to be a significant predictor of Physical and Psychological Well-Being and Self-Perception in self-report questionnaires and of total T-values and Physical Well-Being in proxy questionnaires. However, the associations of Physical Well-Being in both self-report and proxy questionnaires, as well as Psychological Well-Being in self-reports, with burosumab dosage appeared stronger than their associations with Financial Resources.

## Conclusions

In this real-world study of children with XLH under burosumab treatment, HRQoL was comparable to or slightly above German reference values. A burosumab dosage above 0.6 mg/kg every two weeks, which is slightly below the starting dose recommended in the current Summary of Product Characteristics (0.8 mg/kg per two weeks), was associated with improved Physical Well-Being. These findings should not be interpreted as supporting dose escalation to the highest tolerable dose. Rather, they highlight the importance of incorporating HRQoL into treatment decision-making, alongside clinical aspects and biochemical parameters. Our data suggest integrating assessments of HRQoL and its potential predictors, such as burosumab dosage, into individualized treatment monitoring and pharmacological management for burosumab-treated children with XLH. The findings of the present study may be of clinical relevance in patients who, despite adequate biochemical normalization of serum parameters, continue to experience reduced HRQoL while receiving a burosumab dose of less than 0.6 mg/kg every 14 days. In such cases, a cautious dose escalation may be considered, given that the full effects of burosumab on the altered bone metabolism in X-linked hypophosphatemia are not yet fully understood and may not be entirely reflected by conventional laboratory parameters in blood.

## Supplementary Information

Below is the link to the electronic supplementary material.

Supplementary Material 1

Supplementary Material 2

## Data Availability

Some or all datasets generated and/or analyzed during the current study are not publicly available but are available from the corresponding author on reasonable request.
