# Supplementary material for: Health-Related Quality of Life in Children with X-Linked Hypophosphatemia Treated with Burosumab: Real-World Data from a German-Swiss Study
Source: Calcif Tissue Int. 2026 Sep 16;117(1):140. doi: 10.1007/s00223-026-01602-x (PMC13582184; doi:10.1007/s00223-026-01602-x)
Supplement: Supplementary file 1 [file 223_2026_1602_MOESM1_ESM.docx]

**Supplementary material to the article “Health-related quality of life in children with X-linked hypophosphatemia treated with burosumab: real-world data from a German-Swiss study”, published in *Calcified Tissue International***

Ineke Böckmann^a^, Martin Klein^b^, Helena Mutze^b^, Mirko Rehberg^c^, Giuseppina Spartà^d^, Katrina Evers^d^, Karl Peter Schlingmann^e^, Markus J. Kemper^f^, Annette Richter-Unruh^g^, Dorothee Schmidt^h^, Olaf Hiort^h^, Karina Grohmann-Held^i^, Carmen Schröder^i^, Ute Derichs^j^, Clemens Freiberg^k^, Marcus Weitz^l^, Desiree Dunstheimer^m^, Elmar Schmid^n^, Ulrike John-Kroegel^o^, Oliver Metzing^p^, Sabine Heger^q^, Norbert Jorch^r^, Hagen Staude^s^, Ludwig Patzer^t^, Elke Wühl^u^, Carl-Joachim Partsch^v^, Angela Hübner^w^, Michaela Marx^x^, Christof Land^y^, Norbert Albers^z^, Jens Banzer^z^, Inka Baus^aa^, Frauke Wilkening^ab^, Kristina Möller^ac^, Verena Wagner^ad^, Gabriele Krauch^ae^, Michaela Gessner^af^, Alexandra Goischke^ag^, Maren Leifheit-Nestler^a^, Miroslav Zivicnjak^a^, Dirk Schnabel^ah^ and Dieter Haffner^a^ on behalf of the German Society for Pediatric Nephrology (GPN) and the German Society for Pediatric and Adolescent Endocrinology and Diabetology (DGPAED)

**Corresponding author:**

Prof. Dr. Dieter Haffner

Department of Pediatric Kidney, Liver, Metabolic and Neurological Diseases

Hannover Medical School

Carl-Neuberg-Str. 1, D-30625 Hannover

Phone: +49 511 532 3213, Fax: +49 511 532 3911

E-mail: haffner.dieter@mh-hannover.de

ORCiD: 0000-0002-9601-7813

**Supplementary table 1.** T-values of HRQoL in 64 pediatric XLH patients on burosumab treatment rated by patients and caregivers using the KIDSCREEN-52 questionnaire compared to German reference values of healthy children

| **Scale** | **Self-report 8 – 18** | | |  | **Proxy 8 – 18** | | |
| --- | --- | --- | --- | --- | --- | --- | --- |
|  | Patient cohort | Reference values | *p* |  | Patient cohort | Reference values | *p* |
| Physical Well-Being | 52.4 (44.7; 59.4) | 52.4 (42.5; 58.9) | 0.904 |  | 49.5 (41.1; 54.3) | 52.6 (46.3; 56.9) | **0.009** |
| Psychological Well-Being | 51.8 (45.1; 61.5) | 52.3 (41.5; 57.6) | 0.935 |  | 55.3 (48.9; 58.2) | 52.1 (46.6; 58.2) | 0.307 |
| Moods & Emotions | 52.7 (43.9; 62.1) | 49.7 (39.3; 57.4) | 0.122 |  | 46.1 (36.2; 58.0) | 48.6 (41.9; 54.6) | 0.665 |
| Self-Perception | 52.2 (46.1; 64.9) | 49.8 (40.0; 58.9) | 0.052 |  | 46.5 (42.3; 56.2) | 49.1 (44.3; 56.2) | 0.401 |
| Autonomy | 50.8 (44.4; 57.3) | 53.2 (43.3; 58.3) | 0.515 |  | 50.9 (45.7; 57.1) | 53.9 (48.2; 61.0) | **0.041** |
| Parent Relation & Home Life | 51.8 (47.5; 58.5) | 49.5 (39.5; 55.7) | **0.017** |  | 49.4 (46.3; 58.5) | 49.4 (44.5; 55.1) | 0.304 |
| Financial Resources | 62.9 (49.3; 62.9) * | 54.5 (41.9; 62.9) | **0.025** |  | 65.0 (51.9; 65.0) | 55.4 (51.8; 61.8) | **<0.001** |
| Peers & Social Support | 52.4 (47.5; 58.1) | 50.2 (40.4; 54.9) | **0.020** |  | 50.7 (46.4; 56.7) | 50.7 (46.4; 55.4) | 0.548 |
| School Environment | 52.2 (46.9; 58.9) | 50.4 (40.2; 56.7) | **0.047** |  | 52.1 (45.4; 57.0) | 50.3 (45.3; 56.9) | 0.369 |
| Social Acceptance (Bullying) | 58.8 (42.2; 58.8) * | 48.9 (35.5; 58.9) | 0.173 |  | 50.6 (39.3; 58.8) | 50.6 (44.8; 58.8) | 0.432 |
| Total | 52.3 (47.5; 58.0) | 51.1 (40.4; 58.0) | 0.115 |  | 51.4 (44.7; 55.9) | 51.3 (46.0; 57.5) | 0.780 |

Data is presented as median (interquartile range). *p* values ware calculated using one sample Wilcoxon test and unpaired Mann-Whitney test, as appropriate. * p < 0.01 vs T-values in proxy reports.

**Supplementary table 2.** Comparison of T values of 10 dimensions of HRQoL between females and males with XLH treated with burosumab in self-report questionnaires.

| **Characteristics** | **Female (n=29)** | **Male (n=20)** | ***p*** |
| --- | --- | --- | --- |
| Physical Well-Being | 52.4 (42.5 – 59.4) | 52.4 (49.6 – 64.3) | 0.164 |
| Psychological Well-Being | 54.5 (49.3 – 61.5) | 51.8 (45.1 – 54.5) | 0.260 |
| Moods & Emotions | 49.1 (43.9 – 54.0) | 54.0 (49.1 – 62.1) | 0.409 |
| Self-Perception | 55.4 (46.1 – 69.8) | 52.2 (43.2 – 60.1) | 0.465 |
| Autonomy | 53.2 (48.7 – 60.5) | 50.8 (46.9 – 60.5) | 0.946 |
| Parent Relation & Home Life | 54.6 (47.5 – 58.5) | 51.8 (49.5 – 65.9) | 0.696 |
| Financial Resources | 56.3 (49.3 – 62.9) | 62.9 (49.3 – 62.9) | 0.727 |
| Peers & Social Support | 54.9 (46.7 – 58.1) | 52.4 (52.4 – 58.1) | 0.927 |
| School Environment | 52.2 (46.9 – 58.9) | 56.4 (48.6 – 65.9) | 0.504 |
| Bullying | 58.8 (42.2 – 58.8) | 48.1 (42.2 – 58.8) | 0.146 |
| Total | 53.8 (51.1 – 59.3) | 52.8 (50.5 – 57.7) | 0.760 |

Data is presented as median (interquartile range). *p* values ware calculated using unpaired Mann-Whitney test.

**Supplementary table 3.** Comparison of T values of 10 dimensions of HRQoL between females and males with XLH treated with burosumab in proxy questionnaires.

| **Characteristics** | **Female (n=34)** | **Male (n=25)** | ***p*** |
| --- | --- | --- | --- |
| Physical Well-Being | 49.5 (43.7 – 59.4) | 46.5 (41.1 – 52.7) | 0.609 |
| Psychological Well-Being | 55.3 (48.9 – 58.2) | 55.3 (48.9 – 58.2) | 0.994 |
| Moods & Emotions | 48.6 (43.9 – 62.7) | 46.1 (38.0 – 58.0) | 0.503 |
| Self-Perception | 52.3 (42.3 – 56.2) | 46.5 (42.3 – 56.2) | 0.562 |
| Autonomy | 51.0 (48.2 – 57.1) | 51.0 (45.7 – 57.1) | 0.994 |
| Parent Relation & Home Life | 49.4 (46.9 – 58.5) | 49.4 (44.5 – 55.1) | 0.235 |
| Financial Resources | 65.0 (51.9 – 65.0) | 65.0 (51.9 – 65.0) | 0.646 |
| Peers & Social Support | 53.0 (46.4 – 57.9) | 50.7 (44.4 – 55.4) | 0.550 |
| School Environment | 52.1 (45.4 – 57.1) | 52.1 (43.3 – 54.5) | 0.861 |
| Bullying | 58.8 (44.8 – 58.8) | 50.6 (39.3 – 58.8) | 0.693 |
| Total | 52.2 (48.6 – 56.6) | 51.4 (46.3 – 55.6) | 0.434 |

Data is presented as median (interquartile range). *p* values ware calculated using unpaired Mann-Whitney test.

| Characteristics | Burosumab dosage  < 0.6 mg/kg per 14 days | Burosumab dosage  ≥ 0.6 mg/kg per 14 days | p |
| --- | --- | --- | --- |
| n (%) | 30 (46.9) | 34 (53.1) | n.a. |
| Serum phosphate, mmol/l | 0.97 (0.89; 1.11) | 1.01 (0.87; 1.11) | 0.750 |
| Serum phosphate, z-score | -2.45 (-2.90; -1.36) | -2.32 (-3.46; -1.72) | 0.385 |
| Hypophosphatemia, n(%) | 16 (53.3) | 22 (64.7) | 0.597 |
| Serum ALP, U/l | 326.0 (227.3; 451.0) | 316.0 (261.5; 392.5) | 0.623 |
| Serum ALP, z-score | 1.57 (0.58; 3.16) | 1.05 (0.27; 2.61) | 0.458 |
| Serum ALP >2 z-score, n (%) | 12 (40.0) | 11 (32.4) | 0.592 |
| Serum calcium, mmol/l | 2.40 (2.35; 2.49) | 2.43 (2.34; 2.44) | 0.624 |
| Serum calcium, z-score | -0.27 (-1.81; 0.94) | -0.57 (-1.72; 0.05) | 0.292 |
| Serum iPTH, ng/l | 53.6 (37.2; 68.8) | 42.9 (33.3; 51.2) | 0.212 |
| Serum iPTH, z-score | 1.63 (0.63; 2.31) | 1.02 (0.33; 1.51) | 0.212 |
| Serum 1,25(OH)2D, pg/ml | 73.8 (49.5; 80.7) | 76.9 (56.7; 89.4) | 0.258 |
| Serum 1,25(OH)2D, z-score | 0.62 (-1.32; 1.05) | 0.79 (-0.69; 1.56) | 0.258 |
| Serum 25(OH)D, ng/ml | 23.2 (19.7; 28.2) | 20.8 (18.0; 24.5) | 0.504 |

**Supplementary table 4.** Parameters of mineral and bone metabolism in 30 pediatric XLH patients on a burosumab dosage below 0.6 mg/kg given every 14 days and 34 patients on a dosage of or above 0.6 mg/kg per 14 days

Data is presented as median (interquartile range) or n (%), p values were calculated using one sample Wilcoxon test and unpaired Mann-Whitney test, as appropriate. ^a^ p < 0.05 versus healthy children. AP, alkaline phosphatase; PTH, intact parathyroid hormone; 25(OH)D, 25-hydroxyvitamin D; 1,25(OH)2D, 1,25-dihydroxyvitamin D

**Supplementary figure 1.**

Parameters of mineral and vitamin D metabolism in pediatric XLH patients treated with a dosage of <0.6 mg/kg per 14 days (n=30) and ≥0.6 mg/kg per 14 days (n=34). Data are given as age- and sex-related z-scores. Gray shaded areas indicate the normal range, corresponding to mean ±2 z-score. ALP, alkaline phosphatase; iPTH, intact parathyroid hormone; 1,25(OH)_2_D, 1,25-dihydroxyvitamin D.
