# Supplementary material for: Health-Related Quality of Life in Children with X-Linked Hypophosphatemia Treated with Burosumab: Real-World Data from a German-Swiss Study
Source: Calcif Tissue Int. 2026 Sep 16;117(1):140. doi: 10.1007/s00223-026-01602-x (PMC13582184; doi:10.1007/s00223-026-01602-x)
Supplement: Supplementary file 2 [file 223_2026_1602_MOESM2_ESM.docx]

**Scatterplots ALP, phosphate, height z-score**
